# HIV prevention among adolescents travestis and transgender women in three Brazilian capitals, 2019-2023

**DOI:** 10.1590/S2237-96222024v33e2024321.especial.en

**Published:** 2025-01-10

**Authors:** Beo Oliveira Leite, Laio Magno, Dirceu Greco, Alexandre Grangeiro, Ines Dourado

**Affiliations:** 1 Universidade Federal da Bahia, Instituto de Saúde Coletiva, Salvador, BA, Brazil; 2 Universidade do Estado da Bahia, Departamento de Ciências da Vida, Salvador, BA, Brazil; 3 Universidade Federal de Minas Gerais, Escola de Medicina, Belo Horizonte, MG, Brazil; 4 Universidade de São Paulo, Departamento de Medicina Preventiva, São Paulo, SP, Brazil

**Keywords:** Adolescente, Travestis, Mujeres Trans, VIH, Discriminación de Género, Adolescent, Travestis, Transgender Women, HIV, Gender-based discrimination

## Abstract

**Objective:**

To describe HIV prevention strategies and gender-based discrimination among adolescent *travestis* and transgender women.

**Methods:**

This was a cross-sectional study involving 148 adolescent *travestis* and transgender women aged 15 to 19 years in Salvador, Bahia state, São Paulo, São Paulo state, and Belo Horizonte, Minas Gerais state, conducted between February 2019 and March 2023. Fisher’s exact test was performed to assess differences between prevention strategies and gender-based discrimination within healthcare services.

**Results:**

18.9 % reported engaging in receptive anal sex using condom in the past 6 months; 62.2% had undergone HIV test at least once in their lifetime; and 88.5% had started pre-exposure prophylaxis (PrEP) for HIV. Adolescents aged 15 to 17 years with previous experiences of discrimination in healthcare services had undergone fewer HIV tests in their lifetime or had started PrEP less frequently.

**Conclusion:**

There is an urgent need for the implementation of public policies that eradicate gender-based discrimination and expand access to HIV prevention.

## Study contributions

**Main results:**

The primary experiences of discrimination occurred in school and family environments as well as in public spaces. Discrimination within healthcare services was associated with limited condom use and resistance to initiating pre-exposure prophylaxis (PrEP).

**Implications for services:**

Expand access to prevention strategies in this population and develop strategies to combat gender-based discrimination within healthcare services, as well as in family and school environments and in public spaces.

**Perspectives:**

Strategies to provide access to health services must be prioritized, focusing on demand creation and establishing a connection between adolescent *travestis* and transgender women and healthcare services.

## RESUMEN

**Objetivo:**

Describir las prácticas de prevención del VIH y la discriminación de género entre adolescentes travestis y mujeres trans

**Métodos:**

Estudio transversal con 148 adolescentes travestis y mujeres trans de 15 a 19 años de edad en Salvador, São Paulo y Belo Horizonte entre febrero de 2019 y marzo de 2023. Se utilizó la prueba exacta de Fisher para verificar diferencias entre las prácticas de prevención y la discriminación de género en los servicios de salud

**Resultados:**

El 18,9% informó haber tenido relaciones sexuales anales con preservativo en los últimos 6 meses; el 62,2% se había realizado la prueba de VIH alguna vez en la vida; y el 88,5% había iniciado la profilaxis previa a la exposición (PrEP) para el VIH. Las adolescentes de entre 15 y 17 años con experiencias previas de discriminación en los servicios habían realizado menos pruebas de VIH en su vida o habían iniciado PrEP con menor frecuencia

**Conclusión:**

Se enfatiza la urgencia de implementar políticas públicas que erradiquen la discriminación de género y amplíen el acceso a la prevención del VIH.

## INTRODUCTION

Brazil is the country with the highest number of homicides among the transgender population worldwide.^1^ Over the 15 years of data reported on this issue, since the first report carried out in 2009 to the most recent one in 2023, Brazil continues to lead the ranking of deaths among this population.^1^ The majority of these deaths are due to homicide, with victims generally being transgender women (TGW) and *travestis* aged between 18 and 29 years, of Black or mixed-race/skin color, and in situations of high vulnerability.^2^

Violence against *travestis* and TGW can be explained by the stigma and gender-based discrimination (GBD) prevalent throughout society, culturally rooted in cis-heteronormativity.^2^ Although transphobia has been classified as a crime by the Supreme Federal Court since 2019,^3^ discrimination still persists in various contexts: individual, interpersonal and institutional.^4^,^5^

Transphobia is reported in numerous situations, such as disrespect for social names, discrimination in healthcare services or by healthcare professionals, breakdown of family ties, and physical, verbal, or sexual aggression in Brazil^4^,^5^ and other countries.^6^,^7^ *Travestis* and TGW are at a higher risk of sexually transmitted infections, especially HIV.^8^

Globally, HIV prevalence among *travestis* and TGW was 19.1%,^9^ while in Brazil the prevalence ranged between 9% and 40% across different municipalities.^9,10^ Vulnerability to HIV can be attributed to behavioral factors (unprotected sex),^11^ programmatic factors (limited access to services, prevention strategies and technologies^)5^and socio-structural factors (stigma and discrimination).^4,12^

The association between discrimination and HIV infection with HIV prevention strategies, such as HIV testing and condom use, has been described in the literature;^11,12^ however, the focus of these studies has been on the adult *travestis* and TGW. Regarding *travestis* and adolescent TGW, there is a knowledge gap concerning these outcomes.

Adolescence is a phase marked by the onset of biopsychosocial changes. This is a crucial time for health interventions, as behaviors learned during this time may persist into adulthood.^13^ For adolescent *travestis* and TGW, this can also be a critical time, often marked by the beginning of social transition, the use of hormones or other body modification strategies, fragile or broken family ties, and experiences of gender-based discrimination.^14^

Access for adolescent *travestis* and TGW to healthcare services or HIV prevention strategies and other sexually transmitted infections may be hindered by: i) a lack of strategies to engage adolescents with services, qualified professionals to support this population, or a lack of ongoing educational actions for prevention;^15^ or ii) GBD that can operate both interpersonally among healthcare professionals and institutionally through services.^12,16^ This study aimed to investigate the prevalence of HIV, experiences of GBD, and HIV prevention strategies among adolescent *travestis* and TGW in three Brazilian capitals from 2019 to 2023.

## METHODS

### Study design

The PrEP15-19 study is the first cohort study in Latin America aimed at demonstrating the effectiveness of HIV pre-exposure prophylaxis (PrEP) among adolescent men who have sex with men and adolescent *travestis* and TGW, aged 15 to 19, residing in three major Brazilian capitals: Salvador, Bahia state, São Paulo, São Paulo state, and Belo Horizonte, Minas Gerais, state. This is a cross-sectional analysis using baseline data from adolescent *travesti* and TGW recruited between February 2019 and March 2023 in these three capitals.

### Population

Eligible adolescent *travestis* and TGW were those who met the following criteria: self-identified as a *travesti* or TGW; aged 15 to 19 years; residing in one of the cities where the study was conducted; and reported sexual relations with cisgender men, *travestis* or TGW. The exclusion criteria were: being under the influence of alcohol or other drugs at the time of the study or having a mental disorder that could impair participation.

### Data collection

Initial formative research was conducted to identify the social settings for adolescent *travestis* and TGW, and strategies for online and in-person demand generation were developed to reach and recruit this population.^17^ Eligible participants were referred to the project clinics at each site, or received by spontaneous creation. All participants completed a standard questionnaire administered by a trained researcher to collect sociodemographic and sexual behavior information, in a private setting dedicated to this purpose. Third- and fourth-generation rapid HIV-1/2 tests were performed. All participants received pre- and post-test counseling, and were informed of their results. If the first fourth-generation rapid test result was reactive, a second third-generation rapid test was performed following the Ministry of Health’s HIV testing algorithm. If HIV infection was confirmed, participants were immediately referred for clinical follow-up and treatment initiation. More details can be found in the paper by Dourado et al.^15^

### Study variables

The variables described in this study were selected from the questionnaires and organized into the following blocks.

- Sociobehavioral: age (15-17 years , 18-19 years); race/skin color (white, black, brown, indigenous, asian); schooling (elementary education, high school, higher education); housing (lives with parents or guardians, does not live with parents or guardians); employment status (unemployed or unpaid work, paid employment); participation in a civil society organization (never participated, has participated); and age at first sexual intercourse (up to 15 years old, 15 years or older).
- HIV prevention strategies: receptive anal sex using a condom in the past 6 months (no, yes); had undergone HIV test at least once in their lifetime; (no, yes); and initiation of PrEP during the study (no, yes).
- Reports of GBD in the 6 months prior to cohort entry: not selected or dismissed from employment; was poorly served or prevented from entering commercial/leisure establishments; mistreated in healthcare services or by healthcare professionals; mistreated or marginalized by teachers at school/university/course; mistreated or marginalized by peers at school/university/course; excluded or marginalized by friends; excluded or marginalized by neighbors; excluded or marginalized within the family; excluded or marginalized in religious settings; mistreated by police or poorly attended in police stations; mistreated in public services (shelters, local government offices, transport); blackmailed or extorted; felt unsafe walking in public spaces; harassed on social media or other virtual environments; mistreated/discriminated against at work. For each of these situations, responses were “sometimes” or “once,” categorized as “once or more,” and “never.” Responses of “I do not wish to answer” or “not applicable” were disregarded.

### Data analysis

Absolute and relative frequencies were calculated for each variable, stratified by age groups of younger (15-17 years) and older (18-19 years) adolescent *travestis* and TGW. Differences in proportions of HIV prevention strategies were investigated according to reports of discrimination in healthcare services or by healthcare professionals, also stratified by age group. Fisher’s exact test was performed to verify the differences between the proportions considering a descriptive level of statistical significance of the alpha error of 5%. All analyses were performed using R version 4.3.2.

### Ethical aspects

The study was conducted in accordance with Resolution of the National Research Ethics Committee No. 466/2012 and approved by the Research Ethics Committee of the World Health Organization, and by the committees of the Universidade Federal da Bahia on 26/03/2019 (No. 3,224,384/ Certificate of Submission for Ethical Appraisal (CAAE): 89993018.9.3002.5030), Universidade de São Paulo on 12/13/2024 (No. 4,229,488/CAAE: 89993018.9.0000.0065) and Universidade Federal de Minas Gerais on 06/05/2024 (No. 2,027,889/CAAE: 89993018.9.3003.5149). All adolescent *travestis* and TGW aged 18 or over who agreed to participate in the study signed a free and informed consent form. For adolescent *travestis* and TGW under 18 years old, each site followed different procedures. In São Paulo, parental or guardian consent was waived; requiring only the adolescent’s signed assent form. In Salvador, parents or guardians signed the informed consent, but in cases of broken family ties or evidence of violence, the consent requirement was waived. In Belo Horizonte, the signing of the informed consent form by parents or guardians was mandatory in all cases.

## RESULTS

In this study, a total of 148 adolescent *travestis* and TGW were recruited, 29.0% (43) in Salvador, 51.4% (76) in São Paulo and 19.6% (19) in Belo Horizonte. Of these, 31.8% (47) were aged 15 to 17 years, while 68.2% (101) were aged 18 to 19 years (Table 1). The prevalence of HIV in this sample was 2.7% (4/148).

**Table 1:** Sociobehavioral and HIV prevention characteristics among adolescent *travestis* and transgender women in three Brazilian capitals (n=148), 2019-2023

Variables	Total	Age (years)	
		15-17	18-19
	n (%)	n (%)	n (%)
**Age (years)**			
15-17	47 (31.8)		
18-19	101 (62.8)		
**Race/skin color**			
White	37 (25.3)	10 (21.7)	27 (27.0)
Mixed-race	38 (26.0)	12 (26.1)	6 (26.0)
Black	63 (43.2)	26 (50)	40 (40.0)
Asian	2 (1.4)	-	2 (2.0)
Indigenous	6 (4.1)	1 (2,2)	5 (5.0)
**Education level**			
Elementary education	35 (23.6)	18 (38.3)	17 (16.8)
High school	105 (71.0)	29 (61.7)	76 (75.3)
Higher education	8 (5.4)	-	8 (7.9)
**Housing**			
Lives with parents or guardians	85 (63.0)	30 (66.7)	55 (61.1)
Does not live with parents or guardians	50 (37.0)	15 (33.3)	35 (38.9)
**Employment status**			
Unemployed or unpaid work	89 (65.9)	30 (66.7)	59 (65.6)
Paid employment	46 (34.1)	15 (33.3)	31 (34.4)
**Civil society organization**			
Never participated	113 (83.7)	39 (86.7)	74 (82.2)
Has participated	22 (16.3)	6 (13.3)	16 (17.8)
**Age at first sexual intercourse (years)**			
Under 15	90 (67.7)	22 (48.9)	62 (68.9)
15 or older	43 (32.3)	23 (51.1)	28 (31.1)
**Receptive anal sex using a condom in the last 6 months**			
No	120 (81.1)	39 (83.0)	81 (80.2)
Yes	28 (18.9)	8 (17.0)	20 (19.8)
**Has undergone an HIV test at least once**			
No	51 (37.8)	23 (51.1)	28 (31.1)
Yes	84 (62.2)	22 (48.9)	62 (68.9)
**Initiated pre-exposure prophylaxis during the study**			
No	15 (11.5)	8 (20.0)	7 (7.8)
Yes	115 (88.5)	32 (80.0)	83 (92.2)

The majority of adolescent *travestis* and younger TGW (15-17 years old) self-identified as Black (50.0%), were either attending or had completed high school (61.7%), lived with parents or guardians (66.7%), were unemployed or engaged in unpaid work (66.7%), had never participated in a civil society organization (86.7%), had their first sexual intercourse at 15 years or older (51.1%), had never undergone an HIV test (51.1%) and initiated PrEP use during the study (80.0%). In the last 6 months, 17% reported engaging in receptive anal sex using a condom (Table 1).

Among adolescent *travestis* and older TGW (18-19 years old) the majority self-identified as Black (40.0%), were either attending or had completed high school (75.3%), lived with parents or guardians (61.1%), were unemployed or engaged in unpaid work (65.6%), had never participated in a civil society organization (82.2%) and initiated PrEP use during the study (92.2%). Most of them had their first sexual intercourse before the age of 15 (68.9%), 31.1% had never undergone an HIV test and 19.8% engaged in receptive anal sex using a condom in the last 6 months (Table 1).

Table 2 highlights the reports of discrimination by school/college/course peers (53%) and within the family environment (51.1%) and fear of walking in public spaces (71.9%), which were reported by more than half of all adolescent *travestis* and TGW, especially among those aged 18 to 19 (the oldest group): 53.9%, 60.0% and 76.7%. Previous history of discrimination at any time in health services or by healthcare professionals before entering the cohort was reported by 31.8% of the total adolescent *travesti* and TGW, 26.7% among the youngest group and 34.4% among the oldest group (Table 2).

**Table 2:** Experience of discrimination in the last 6 months prior to cohort entry among adolescent *travestis* and transgender women in the three Brazilian capitals (n=148), 2019-2023

Discrimination	Total	Age (years)	
		15-17	18-19
	n (%)	n (%)	n (%)
**Not selected for a job or dismissed from employment**			
Never	74 (60.2)	27 (65.9)	47 (57.3)
Once or more	49 (39.8)	14 (34.1)	35 (42.7)
**Received poor service or was barred from entering shops/leisure venues**			
Never	71 (53.0)	29 (64.4)	42 (47.2)
Once or more	63 (47.0)	16 (35.6)	47 (52.8)
**Received poor service in healthcare settings or from health professionals**			
Never	92 (68.2)	33 (73.3)	59 (65.6)
Once or more	43 (31.8)	12 (26.7)	31 (34.4)
**Mistreated or marginalized by teachers at school/college/course?**			
Never	91 (61.9)	31 (68.9)	60 (67.4)
Once or more	43 (32.1)	14 (31.1)	29 (32.6)
**Mistreated or marginalized by peers at school/college/course?**			
Never	63 (47.0)	22 (48.9)	41 (46.1)
Once or more	71 (53.0)	23 (51.1)	48 (53.9)
**Excluded or marginalized from a group of friends**			
Never	77 (57.0)	27 (60.0)	50 (55.6)
Once or more	58 (43.0)	18 (40.0)	40 (44.4)
**Excluded or marginalized by neighbors**			
Never	72 (53.7)	28 (62.2)	44 (49.4)
Once or more	62 (46.3)	17 (37.8)	45 (50.6)
**Excluded or marginalized in their family environment**			
Never	66 (48.9)	30 (66.7)	36 (40)
Once or more	69 (51.1)	15 (33.3)	54 (60)
**Excluded or marginalized in a religious environment**			
Never	77 (57.5)	26 (57.8)	51 (57.3)
Once or more	57 (42.5)	19 (42.2)	38 (42.7)
**Mistreated by police officers or poorly served at police stations**			
Never	95 (70.4)	32 (71.1)	63 (70.0)
Once or more	40 (29.6)	13 (28.9)	27 (30.0)
**Mistreated in public services (shelters, local authorities, transport)**			
Never	96 (71.1)	34 (75.6)	62 (68.9)
Once or more	39 (28.9)	11 (24.4)	28 (31.1)
**Blackmailed or extorted for money**			
Never	123 (91.1)	40 (88.9)	83 (92.2)
Once or more	12 (8.9)	5 (11.1)	7 (7.8)
**Felt afraid to walk in public spaces**			
Never	38 (28.1)	17 (37.8)	21 (23.3)
Once or more	91 (71.9)	28 (62.2)	69 (76.7)
**Harassed on social media or other virtual environments**			
Never	72 (53.3)	25 (55.6)	47 (52.2)
Once or more	63 (46.7)	20 (44.4)	43 (47.8)
**Mistreated/discriminated at work**			
Never	95 (71.4)	36 (80.0)	59 (67.1)
Once or more	38 (28.6)	9 (20.0)	29 (32.9)

**Figure 1:**
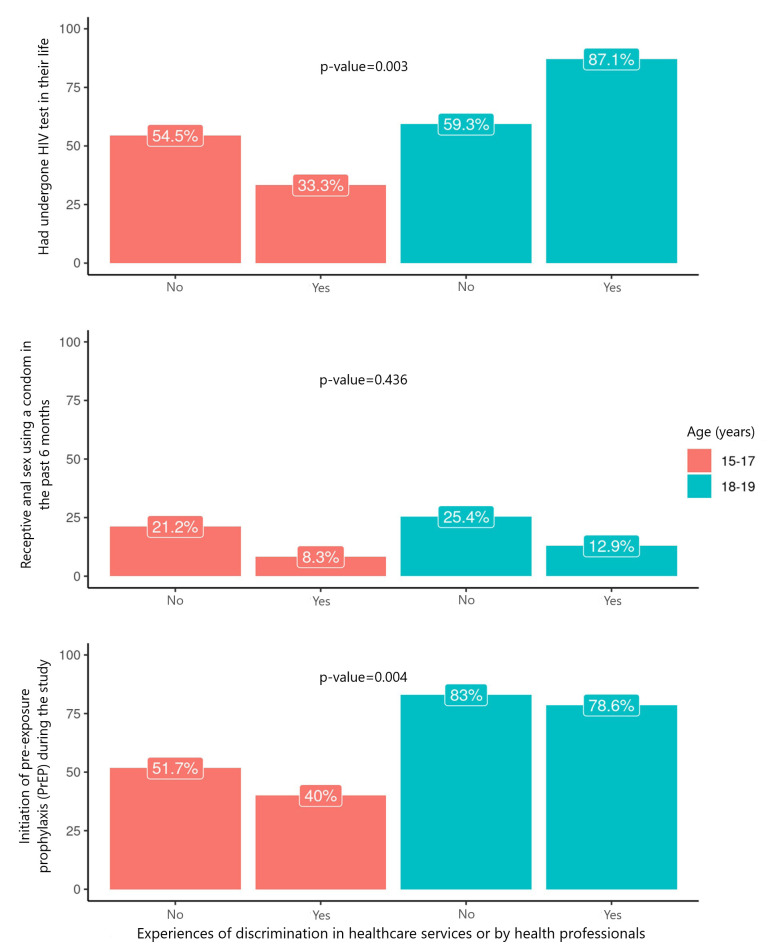
HIV prevention strategies according to experiences of discrimination in healthcare services or by healthcare professionals among adolescent *travestis* and transgender women in the three Brazilian capitals (N=148), 2019-2023

It is noteworthy that, among *travesti* adolescents and TGW who had undergone HIV test at least once in their lifetime (p-value 0.003), or initiated PrEP use (p-value 0.004), experiences of discrimination in healthcare services or by health professionals prior to cohort entry was greater among the oldest group (87.1% and 78.6%) compared to the youngest group (33.3% and 40.0%) (Figure 1).

## DISCUSSION

This study identified a high prevalence of HIV among adolescent *travestis* and TGW (2.7%) when compared to the cisgender female population (0.4%).^18^ Notably, nearly half of the adolescent *travestis* and TGW reported never having undergone an HIV test, particularly the youngest participants, when evaluating sexual prevention strategies.

The prevalence among *travestis* and TGW reached 25.0% in Porto Alegre,^19^ 31.2% in Rio de Janeiro^20^ and 24.3% in Salvador.^10^ The prevalence of HIV among adolescent *travestis* and TGW observed in this study is relatively low when compared to the adult group. This may be related to the fact that adolescents may have had little time between the onset of sexual activity and potential exposure to the risk of infection. It is noteworthy that, during the study period (February 2019 to March 2023), the COVID-19 pandemic led to a reduction in the number of sexual contacts observed in this population.^21^

In Brazil, a study conducted in Rio de Janeiro revealed that younger *travestis* and TGW, aged 18 to 24 years, were less likely to have ever undergone an HIV test (OR 0.4; 95%CI 0.2;0.7) compared to those older than 24 years.^24^

Access to HIV testing remains inadequate for the trans population. Despite the expansion of HIV testing services in primary healthcare, not all services and healthcare professionals are prepared to comprehensively attend to *travestis* and TGW.^5,16^ HIV testing is still largely concentrated in specialized services, which are few and do not provide effective and adequate access to this population.^25^

Receptive anal sex using a condom was rarely observed in this study. In Salvador, the prevalence of receptive anal sex using a condom in the last 6 months in 2016 and in the last 30 days in 2017 was 30.7% among *travestis* and TGW aged 18 years or older and 25.2% among *travestis* and TGW aged 15 years or older.^12^ In Rio de Janeiro, the frequency of receptive anal sex using a condom with the last 3 partners among *travestis* and TGW aged 18 years or older was 31.8%.^20^ For young *travestis* and TGW aged 18 to 24 years old, there was a higher likelihood of engaging in condomless receptive anal sex with the last three partners compared to those older than 24 years (OR 1.8; 95%CI 1.1;3.0).^24^

Several factors may explain the low frequency of condom use during receptive anal sex among adolescent *travestis* and TGW compared to the general population: gender-based discrimination and limited access to healthcare services, which includes HIV prevention and care services;^11,12^ lack of guidance or counseling on sexual education;^5,16^ involvement in sex work;^4^ low self-esteem; and limited or no autonomy in deciding to use a condom during sexual intercourses.^26^

Despite the low frequencies of lifetime HIV testing and condom use during receptive anal sex among *travesti* adolescents and TGW, the initiation of daily oral PrEP use in this cohort was high, especially when compared to initiation rates among *travestis* and trans women. In Rio de Janeiro, a study involving *travestis* and adult TGW revealed a PrEP initiation rate of 48%.^27^ The high frequency of initiation of PrEP use among adolescent *travesti* and TGW can be explained by their motivation for prevention due to their vulnerability. Additionally, the provision of youth-friendly services staffed by professionals trained to offer effective counseling may have contributed to this result. This may indicate that PrEP may be a valuable strategy for protection which is already highly vulnerable to HIV infection.^27,28^ In 2022, the update of the Clinical Protocols and Therapeutic Guidelines for PrEP use were updated to expand access to adolescents aged 15 years and older.^29^

It is important emphasizing that the experience of gender-based discrimination reported by adolescent *travesti* and TGW in health services or by professionals in these services pose a barrier to access and adherence to combined prevention strategies, such as HIV testing and the use of PrEP. Even though the present study created welcoming services specifically aimed at the young transgender participants, previous experiences of discrimination in other services may hinder the breaking of internalized barriers among adolescent TGW in establishing trust with healthcare professionals or the service itself.^12,27,30^

This study had some limitations. The sample size and convenience sampling hinder the application of more robust methodologies for determining the magnitude of effects between the variables studied. However, the sample size did not pose a barrier, as the study’s intent is descriptive, and its findings align with those reported in the literature.

While there is motivation to seek prevention strategies, gender-based discrimination, especially in the context of health care for adolescent transgender women, is one of the main obstacles for establishing effective and ethical health care. As a result, access to HIV prevention, such as regular testing, early diagnosis or the use of preventive methods, such as access to condoms or PrEP, are limited. For adolescents, the absence of preventive behaviors and attitudes may persist throughout their lives.

It is necessary to eradicate gender-based discrimination in healthcare and ensure adequate access for the transgender population from adolescence onwards. Key actions include: expanding healthcare coverage for adolescent TGW within the Brazilian National Health System beyond specialized outpatient clinics; investing in demand creation through initiatives that attract younger populations; and ensuring service linkage by establishing welcoming spaces within the Brazilian National Health System.
