# Supplementary material for: HIV prevention among adolescents travestis and transgender women in three Brazilian capitals, 2019-2023
Source: Epidemiol Serv Saude. 2025 Jan 10;33(spe1):e2024321. doi: 10.1590/S2237-96222024v33e2024321.especial.en (PMC11734661; doi:10.1590/S2237-96222024v33e2024321.especial.en)
Supplement: Translation

# Translation: Prevenção do HIV e discriminação de gênero entre adolescentes travestis e mulheres trans em três capitais do Brasil no período 2019-2023

**DOI:** 10.1590/S2237-96222024v33e2024321.especial.pt

**Authors:** Beo Oliveira Leite, Laio Magno, Dirceu Greco, Alexandre Grangeiro, Ines Dourado

**Affiliations:** 1 Universidade Federal da Bahia, Instituto de Saúde Coletiva, Salvador, BA, Brasil; 2 Universidade do Estado da Bahia, Departamento de Ciências da Vida, Salvador, BA, Brasil; 3 Universidade Federal de Minas Gerais, Escola de Medicina, Belo Horizonte, MG, Brasil; 4 Universidade de São Paulo, Departamento de Medicina Preventiva, São Paulo, SP, Brasil

**Keywords:** Adolescente, Travestis, Mulheres Transexuais, HIV, Discriminação de Gênero

## Abstract

**Objetivo:**

Descrever as práticas de prevenção do HIV e a discriminação de gênero entre adolescentes travestis e mulheres trans.

**Métodos:**

Estudo transversal com 148 adolescentes travestis e mulheres trans de 15 a 19 anos de idade em Salvador, São Paulo e Belo Horizonte entre fevereiro/2019 e março/2023. Teste exato de Fisher foi utilizado para verificar diferenças entre práticas de prevenção e discriminação de gênero nos serviços de saúde.

**Resultados:**

18,9% relataram sexo anal com preservativo nos últimos 6 meses; 62,2% realizaram teste para o HIV alguma vez na vida; e 88,5% iniciaram a profilaxia pré-exposição (PrEP) ao HIV. As adolescentes entre 15 e 17 anos com experiências prévias de discriminação nos serviços haviam realizado menos testes de HIV na vida ou iniciaram menos a PrEP.

**Conclusão:**

Ressalta-se a urgência para a implementação de políticas públicas que erradiquem a discriminação de gênero e ampliem o acesso à prevenção ao HIV.

## Contribuições do estudo

**Principais resultados:**

As principais experiências de discriminação ocorreram nos ambientes escolar e familiar e nos espaços públicos. A discriminação nos serviços de saúde esteve relacionada ao limitado uso do preservativo e à resistência ao início do uso da profilaxia pré-exposição (PrEP).

**Implicações para os serviços:**

Ampliar o acesso às estratégias de prevenção nessa população e desenvolver estratégias de combate à discriminação de gênero nos serviços de saúde, assim como nos ambientes familiar e escolar e nos espaços públicos.

**Perspectivas:**

Devem ser priorizadas estratégias para proporcionar acesso aos serviços de saúde, com criação de demanda e vinculação das adolescentes travestis e mulheres trans aos serviços de saúde.

## INTRODUÇÃO

O Brasil é o país com o maior número de homicídios na população trans em todo o mundo.^1^ Nos 15 anos de levantamento de dados quanto ao tema, desde o primeiro relatório realizado em 2009 até o último realizado em 2023, o Brasil segue liderando o *ranking* de mortes dessa população.^1^ As mortes são majoritariamente por homicídio, e as vítimas são em geral travestis e mulheres trans entre 18 e 29 anos de idade, pretas ou pardas, e em situação de alta vulnerabilidade.^2^

A violência contra as travestis e mulheres trans pode ser explicada pelo estigma e pela discriminação de gênero presentes em toda a sociedade, culturalmente enraizado pela cisheteronormatividade.^2^ Apesar de a transfobia ser considerada crime pelo Supremo Tribunal Federal desde 2019,^3^ ainda existe discriminação nos diferentes contextos: individual, interpessoal e institucional.^4,5^

A transfobia é denunciada nas mais diversas situações, tais como desrespeito ao nome social, discriminação nos serviços de saúde ou por profissionais de saúde, rompimento de vínculos familiares e agressão física, verbal ou sexual no Brasil^4 ,5^ e em outros países.^6,7^ As travestis e mulheres trans correm maior risco de infecções sexualmente transmissíveis, especialmente do HIV.^8^

Globalmente, a prevalência para o HIV entre as travestis e mulheres trans foi de 19,1%,^9^ enquanto no Brasil a prevalência variou entre 9% e 40% para diferentes municípios.^9,10^ A vulnerabilidade para o HIV pode ser explicada por fatores comportamentais (sexo desprotegido),^11^ programáticos (menor acesso aos serviços, estratégias e tecnologias de prevenção)^5^ e socioestruturais (o estigma e a discriminação).^4,12^

A associação entre a discriminação e a infecção pelo HIV com as práticas de prevenção ao HIV, tais como a testagem para o HIV e o uso do preservativo, já é descrita na literatura, ^11,12^ contudo o foco desses estudos tem sido a população de travestis e mulheres trans adulta. Em relação às adolescentes travestis e mulheres trans, existe uma lacuna do conhecimento desses desfechos. 

A adolescência é uma fase marcada pelo início de mudanças biopsicossociais. Esse é um momento fundamental para intervenções em saúde, uma vez que os comportamentos aprendidos podem permanecer até a fase adulta.^13^ Para as adolescentes travestis e mulheres trans, esse também pode ser um momento crítico, marcado muitas vezes pelo início da transição social, pelo uso de hormônios ou outras estratégias de modificação corporal, pelos vínculos familiares frágeis ou rompidos e pelo contato com experiências de discriminação de gênero.^14^

O acesso para adolescentes travestis e mulheres trans aos serviços de saúde ou às estratégias de prevenção ao HIV e outras infecções sexualmente transmissíveis pode ser dificultado por: i) falta de estratégias que captem adolescentes para os serviços, profissionais qualificados que acolham essa população ou ausência de ações educativas continuadas para prevenção;^15^ ou ii) discriminação de gênero que pode operar tanto interpessoalmente entre profissionais de saúde quanto institucionalmente através dos serviços.^12,16^ Este estudo teve por objetivo investigar a prevalência de HIV, as experiências de discriminação de gênero e as práticas de prevenção ao HIV entre as adolescentes travestis e mulheres trans em três capitais do Brasil no período 2019-2023. 

## MÉTODOS

### Desenho do estudo

O estudo PrEP15-19 é a primeira coorte da América Latina com o intuito de demonstrar a efetividade da profilaxia pré-exposição (PrEP) ao HIV entre adolescentes homens que fazem sexo com outros homens e adolescentes travestis e mulheres trans, de 15 a 19 anos de idade, residentes de três grandes capitais brasileiras: Salvador, São Paulo e Belo Horizonte. Esta análise é um recorte transversal descritivo com dados da linha de base das adolescentes travestis e mulheres trans recrutadas entre fevereiro de 2019 e março de 2023 nessas três capitais.

### População

As adolescentes travestis e mulheres trans elegíveis foram aquelas que apresentaram os seguintes critérios: autodeclarar-se travesti ou mulher trans; ter entre 15 e 19 anos de idade; residir em uma das cidades de realização do estudo; e ter relações sexuais com homens cisgênero, travestis ou mulheres trans. Os critérios de exclusão foram: estar sob influência de álcool ou de outras drogas no momento da realização do estudo ou ter algum transtorno mental que pudesse comprometer a participação. 

### Coleta dos dados

Pesquisa formativa inicial foi realizada para mapear os locais de socialização das adolescentes travestis e mulheres trans, e estratégias de criação de demandas *online* e presenciais foram desenvolvidas para alcançar e recrutar essa população.^17^ As participantes elegíveis foram encaminhadas às clínicas do projeto de cada sítio, ou recepcionadas por demanda espontânea. Todas responderam a um questionário padrão conduzido pelo pesquisador para coleta de informações sociodemográficas e de comportamento sexual, em um espaço reservado exclusivamente para esse fim. Testes rápidos de 3ª e 4ª geração para o HIV-1/2 foram realizados. Todas as participantes receberam aconselhamento e orientação pré-teste e pós-teste, bem como seus resultados. Se os resultados do primeiro teste rápido de 4ª geração fossem reativos, um segundo teste rápido de 3ª geração seria realizado seguindo o algoritmo para testagem do HIV do Ministério da Saúde. Se a infecção pelo HIV se confirmasse, as participantes seriam encaminhadas imediatamente para o devido acompanhamento clínico e o início do tratamento. Mais detalhes podem ser encontrados no artigo que descreve a metodologia do PrEP15-19.^15^

### Variáveis do estudo

As variáveis a serem descritas neste estudo foram selecionadas a partir dos questionários e organizadas nos blocos a seguir.

- Sociocomportamentais: idade (15-17, anos, 18-19 anos); raça/cor da pele (branca, preta, parda, indígena, amarela); escolaridade (fundamental, Médio, superior); moradia (mora com pais ou responsáveis , não mora com pais ou responsáveis); trabalho (não possui trabalho ou trabalha sem remuneração, possui trabalho com remuneração); participa de organização da sociedade civil (nunca participou, já participou); e idade na primeira relação sexual (menor de 15 anos, 15 anos ou mais).
- Práticas de prevenção do HIV: praticou sexo anal receptivo com preservativo nos últimos 6 meses (não, sim); realizou teste para o HIV na vida (não, *s*im); e iniciou o uso da PrEP no estudo (não, sim).
- Relato de experiências de discriminação dos últimos 6 meses anteriores à entrada na coorte: não foi selecionada ou foi demitida do emprego; foi mal atendida ou impedida de entrar em comércio/locais de lazer; foi mal atendida em serviços de saúde ou por profissionais de saúde; foi maltratada ou marginalizada por professores na escola/faculdade/curso; foi maltratada ou marginalizada por colegas na escola/faculdade/curso; foi excluída ou marginalizada de grupo de amigos; foi excluída ou marginalizada por vizinhos; foi excluída ou marginalizada em seu ambiente familiar; foi excluída ou marginalizada em ambiente religioso; foi maltratada por policiais ou mal atendida em delegacias; foi maltratada em serviços públicos (albergues, subprefeituras, transporte); foi chantageada ou sofreu extorsão de dinheiro; sentiu medo de caminhar em espaços públicos; foi hostilizada nas redes sociais ou em outros ambiente virtuais; foi maltratada/discriminada no seu trabalho. Para cada uma das situações as respostas foram “algumas vezes” ou “uma vez”, categorizado em “uma vez ou mais”, e “nenhuma vez”. Respostas para “não quero responder” ou “não se aplica” foram ignoradas.

### Análises dos dados

Foram calculadas as frequências absolutas e relativas para cada uma das variáveis e segundo os estratos da idade das adolescentes travestis e mulheres trans mais novas (15-17 anos) e mais velhas (18-19 anos). Investigou-se a diferença das proporções do bloco de práticas de prevenção do HIV segundo o relato de discriminação em serviços de saúde ou por profissionais de saúde, também de acordo com os estratos da idade. O teste exato de Fisher foi utilizado para verificar as diferenças entre as proporções considerando um nível descritivo de significância estatística do erro tipo alfa de 5%. Todas as análises foram realizadas em linguagem R versão 4.3.2. 

### Aspectos éticos

O estudo foi desenvolvido de acordo com a Resolução da Comissão Nacional de Ética em Pesquisa nº 466/2012 e aprovado pelo Comitê de Ética em Pesquisa da Organização Mundial da Saúde, e pelos comitês da Universidade Federal da Bahia em 26/03/2019 (nº 3.224.384/ Certificado de Apresentação de Apreciação Ética (CAAE): 89993018.9.3002.5030), Universidade de São Paulo em 13/12/2024 (nº 4.229.488/ CAAE: 89993018.9.0000.0065) e Universidade Federal de Minas Gerais em 06/05/2024 (nº 2.027.889/ CAAE: 89993018.9.3003.5149).Todas as adolescentes travestis e mulheres trans com 18 anos ou mais que aceitaram participar do estudo assinaram o termo de consentimento livre e esclarecido. Para as adolescentes travestis e mulheres trans com menos de 18 anos, cada sítio seguiu diferentes protocolos. Em São Paulo, a assinatura do termo de consentimento livre e esclarecido por pais e responsáveis foi dispensada, sendo necessária apenas a assinatura do termo de assentimento livre e esclarecido pelas adolescentes. Em Salvador, pais ou responsáveis assinavam o termo de consentimento livre e esclarecido, entretanto, em casos de constatação de vínculos familiares rompidos e presença de violência, a assinatura do termo de consentimento livre e esclarecido era liberada. Em Belo Horizonte, a assinatura do termo de consentimento livre e esclarecido por pais ou responsáveis foi mandatória para todos os casos.

## RESULTADOS

Neste estudo foram recrutadas 148 adolescentes travestis e mulheres trans, sendo 29,0% (43) em Salvador, 51,4% (76) em São Paulo e 19,6% (19) em Belo Horizonte. Do total, 31,8% (47) tinham entre 15 e 17 anos de idade, enquanto 68,2% (101) tinham entre 18 e 19 anos de idade (Tabela 1). A prevalência de HIV nesta amostra foi de 2,7% (4/148).

**Tabela 1:** Características sociocomportamentais e de prevenção do HIV entre as adolescentes travestis e mulheres trans das três capitais brasileiras (n=148), 2019-2023

Variáveis	Total	Idade (anos)	
		15-17	18-19
	n (%)	n (%)	n (%)
**Idade (anos)**			
15-17	47 (31,8)		
18-19	101 (62,8)		
**Raça/cor da pele**			
Branca	37 (25,3)	10 (21,7)	27 (27,0)
Parda	38 (26,0)	12 (26,1)	6 (26,0)
Preta	63 (43,2)	26 (50)	40 (40,0)
Amarela	2 (1,4)	-	2 (2,0)
Indígena	6 (4,1)	1 (2,2)	5 (5,0)
**Escolaridade**			
Ensino fundamental	35 (23,6)	18 (38,3)	17 (16,8)
Ensino médio	105 (71,0)	29 (61,7)	76 (75,3)
Ensino superior	8 (5,4)	-	8 (7,9)
**Moradia**			
Mora com pais ou responsáveis	85 (63,0)	30 (66,7)	55 (61,1)
Não mora com pais ou responsáveis	50 (37,0)	15 (33,3)	35 (38,9)
**Trabalho**			
Não possui trabalho ou trabalha sem remuneração	89 (65,9)	30 (66,7)	59 (65,6)
Possui trabalho com remuneração	46 (34,1)	15 (33,3)	31 (34,4)
**Organização da sociedade civil**			
Nunca participou	113 (83,7)	39 (86,7)	74 (82,2)
Já participou	22 (16,3)	6 (13,3)	16 (17,8)
**Idade na primeira relação sexual (anos)**			
Menor de 15	90 (67,7)	22 (48,9)	62 (68,9)
15 ou mais	43 (32,3)	23 (51,1)	28 (31,1)
**Sexo anal receptivo com preservativo nos últimos 6 meses**			
Não	120 (81,1)	39 (83,0)	81 (80,2)
Sim	28 (18,9)	8 (17,0)	20 (19,8)
**Realizou teste para HIV na vida**			
Não	51 (37,8)	23 (51,1)	28 (31,1)
Sim	84 (62,2)	22 (48,9)	62 (68,9)
**Iniciou o uso da profilaxia pré-exposição no estudo**			
Não	15 (11,5)	8 (20,0)	7 (7,8)
Sim	115 (88,5)	32 (80,0)	83 (92,2)

A maioria das adolescentes travestis e mulheres trans mais novas (15-17 anos) se autodeclararam como pretas (50,0%), estavam cursando ou possuíam ensino médio completo (61,7%), moravam com pais ou responsáveis (66,7%), não trabalhavam ou possuíam trabalho sem renda (66,7%), nunca haviam participado de organização da sociedade civil (86,7%), tiveram sua primeira relação sexual com 15 anos de idade ou mais (51,1%), nunca haviam realizado teste para o HIV na vida (51,1%) e iniciaram o uso da PrEP durante o projeto (80,0%). Nos últimos 6 meses, 17% praticaram sexo anal receptivo com preservativo (Tabela 1). 

A maioria das adolescentes travestis e mulheres trans mais velhas (18-19 anos) se autodeclarou preta (40,0%), estava cursando ou finalizou o ensino médio (75,3%), morava com pais ou responsáveis (61,1%), não trabalhava ou possuía trabalho não remunerado (65,6%), nunca participou de organização da sociedade civil (82,2%) e iniciou o uso da PrEP no projeto (92,2%). A maioria delas teve sua primeira relação sexual com menos de 15 anos de idade (68,9%), 31,1% nunca haviam realizado teste para o HIV na vida e 19,8% praticaram sexo anal receptivo com preservativo nos últimos 6 meses (Tabela 1). 

Na Tabela 2, destacam-se os relatos de discriminação por colegas de escola/ faculdade/curso (53%) e no ambiente familiar (51,1%) e do medo de caminhar em espaços públicos (71,9%), declarado por mais da metade do total das adolescentes travestis e mulheres trans, especialmente entre aquelas de 18 a 19 anos (as mais velhas): 53,9%, 60,0% e 76,7%. História prévia de discriminação em qualquer período da vida nos serviços de saúde ou praticada por profissionais de saúde antes da entrada na coorte foi relatada em 31,8% do total das adolescentes travestis e mulheres trans, 26,7% entre as mais novas e 34,4% entre as mais velhas (Tabela 2).

**Tabela 2:** Experiência de discriminação nos últimos 6 meses da entrada na coorte das adolescentes travestis e mulheres trans das três capitais brasileiras (n=148), 2019-2023

Discriminação	Total	Idade (anos)	
		15-17	18-19
	n (%)	n (%)	n (%)
**Não foi selecionada ou foi demitida do emprego**			
Nenhuma vez	74 (60,2)	27 (65,9)	47 (57,3)
Uma vez ou mais	49 (39,8)	14 (34,1)	35 (42,7)
**Foi mal atendida ou impedida de entrar em comércio/locais de lazer**			
Nenhuma vez	71 (53,0)	29 (64,4)	42 (47,2)
Uma vez ou mais	63 (47,0)	16 (35,6)	47 (52,8)
**Foi mal atendido(a) em serviços de saúde ou por profissionais de saúde**			
Nenhuma vez	92 (68,2)	33 (73,3)	59 (65,6)
Uma vez ou mais	43 (31,8)	12 (26,7)	31 (34,4)
**Foi maltratada ou marginalizada por professores na escola/faculdade/curso**			
Nenhuma vez	91 (61,9)	31 (68,9)	60 (67,4)
Uma vez ou mais	43 (32,1)	14 (31,1)	29 (32,6)
**Foi maltratada ou marginalizada por colegas na escola/faculdade/curso**			
Nenhuma vez	63 (47,0)	22 (48,9)	41 (46,1)
Uma vez ou mais	71 (53,0)	23 (51,1)	48 (53,9)
**Foi excluída ou marginalizada de grupo de amigos**			
Nenhuma vez	77 (57,0)	27 (60,0)	50 (55,6)
Uma vez ou mais	58 (43,0)	18 (40,0)	40 (44,4)
**Foi excluída ou marginalizada por vizinhos**			
Nenhuma vez	72 (53,7)	28 (62,2)	44 (49,4)
Uma vez ou mais	62 (46,3)	17 (37,8)	45 (50,6)
**Foi excluída ou marginalizada em seu ambiente familiar**			
Nenhuma vez	66 (48,9)	30 (66,7)	36 (40)
Uma vez ou mais	69 (51,1)	15 (33,3)	54 (60)
**Foi excluída ou marginalizada em ambiente religioso**			
Nenhuma vez	77 (57,5)	26 (57,8)	51 (57,3)
Uma vez ou mais	57 (42,5)	19 (42,2)	38 (42,7)
**Foi maltratada por policiais ou mal atendida em delegacias**			
Nenhuma vez	95 (70,4)	32 (71,1)	63 (70,0)
Uma vez ou mais	40 (29,6)	13 (28,9)	27 (30,0)
**Foi maltratada em serviços públicos (albergues, subprefeituras, transporte)**			
Nenhuma vez	96 (71,1)	34 (75,6)	62 (68,9)
Uma vez ou mais	39 (28,9)	11 (24,4)	28 (31,1)
**Foi chantageada ou sofreu extorsão de dinheiro**			
Nenhuma vez	123 (91,1)	40 (88,9)	83 (92,2)
Uma vez ou mais	12 (8,9)	5 (11,1)	7 (7,8)
**Sentiu medo de caminhar em espaços públicos**			
Nenhuma vez	38 (28,1)	17 (37,8)	21 (23,3)
Uma vez ou mais	91 (71,9)	28 (62,2)	69 (76,7)
**Foi hostilizada nas redes sociais ou em outros ambientes virtuais**			
Nenhuma vez	72 (53,3)	25 (55,6)	47 (52,2)
Uma vez ou mais	63 (46,7)	20 (44,4)	43 (47,8)
**Foi maltratada/discriminada no seu trabalho**			
Nenhuma vez	95 (71,4)	36 (80,0)	59 (67,1)
Uma vez ou mais	38 (28,6)	9 (20,0)	29 (32,9)

Destaca-se que, entre as adolescentes travestis e mulheres trans que realizaram testes para o HIV na vida (p-valor 0,003), ou iniciaram o uso da PrEP no (p-valor 0,004), a experiência de discriminação em serviços de saúde ou por profissionais de saúde sofridas antes da entrada na coorte foi maior entre as mais velhas (87,1% e 78,6%) em comparação com as mais novas (33,3% e 40,0%) (Figura 1).

**Figura 1:** Práticas de prevenção do HIV segundo a experiência de discriminação nos serviços de saúde ou por profissionais de saúde entre as adolescentes travestis e mulheres trans das três capitais brasileiras (n=148), 2019-2023

## DISCUSSÃO

Este estudo identificou a alta prevalência de HIV entre as adolescentes travestis e mulheres trans (2,7%) quando comparada à população de mulheres cis (0,4%).^18^ Nota-se que, entre as práticas de prevenção sexual avaliadas, quase metade das adolescentes travestis e mulheres trans relatou nunca ter realizado teste para HIV ao longo da vida, especialmente as mais jovens. 

As prevalências entre as travestis e mulheres trans chegaram a 25,0% em Porto Alegre,^19^ 31,2% no Rio de Janeiro^20^ e 24,3% em Salvador.^10^ A prevalência do HIV entre as adolescentes travestis e mulheres trans deste estudo é relativamente baixa quando comparada com as adultas. Isso pode estar relacionado ao fato de que as adolescentes podem ter tido pouco tempo entre o início da vida sexual e a eventual exposição com risco para infecção. Ressalta-se que, no período do estudo (fevereiro de 2019 a março de 2023), a pandemia de covid-19 foi responsável pela redução do número de contatos sexuais observado para esta população.^21^

No Brasil, estudo realizado na cidade do Rio de Janeiro revelou que travestis e mulheres trans mais jovens, entre 18 e 24 anos de idade, tinham menor chance de ter realizado teste HIV na vida (OR 0,4; IC_95_% 0,2;0,7) em relação às travestis e mulheres trans com mais de 24 anos idade.^24^

O acesso para a realização de testagem para o HIV permanece precário para a população trans. Mesmo com a expansão de testagem para o HIV na Atenção Primária à Saúde, nem todos os serviços e profissionais de saúde estão preparados para recepcionar e acolher travestis e mulheres trans de maneira integral.^5,16^ A testagem para o HIV ainda se concentra majoritariamente nos serviços especializados, que são poucos e não oferecem acesso efetivo e adequado para essa população.^25^

A prática de sexo anal receptivo com preservativo foi pouco constada neste estudo. Em Salvador, as prevalências de sexo anal receptivo com preservativo nos últimos 6 meses, em 2016, e nos últimos 30 dias, em 2017, foram de 30,7% entre as travestis e mulheres trans com 18 anos ou mais e de 25,2% entre as travestis e mulheres trans de 15 anos ou mais.^12^ Na cidade do Rio de Janeiro, a frequência de sexo anal receptivo com preservativo com os últimos 3 parceiros entre as travestis e mulheres trans com 18 anos ou mais foi de 31,8%.^20^ Para as travestis e mulheres trans jovens entre 18 e 24 anos, estimou-se maior chance de práticas de sexo anal sem preservativo com os últimos 3 parceiros em relação àquelas com mais de 24 anos de idade (OR 1,8; IC_95_%1,1;3,0).^24^

Diversos fatores podem explicar a baixa frequência de sexo anal receptivo com preservativo em adolescentes travestis e mulheres trans em relação à população geral: a discriminação de gênero e o menor acesso a serviços de saúde, o que inclui os serviços de prevenção e atenção ao HIV;^11,12^ a falta de orientação ou aconselhamento para a educação sexual;^5,16^ o trabalho sexual;^4^ a baixa autoestima; e a menor ou nenhuma autonomia em relação à decisão do uso do preservativo nas relações sexuais.^26^

Apesar das baixas frequências encontradas para a realização da testagem para o HIV na vida, e sexo anal receptivo com preservativo entre as adolescentes travestis e mulheres trans, o início do uso da PrEP oral diária na coorte foi alto, especialmente quando comparado com o início do uso por travestis e mulheres trans adultas. Na cidade do Rio de Janeiro, estudo com travestis e mulheres trans adultas revelou o início do uso da PrEP de 48%.^27^ A alta frequência de início do uso da PrEP entre adolescentes travestis e mulheres trans pode ser explicada pela motivação para a prevenção por estarem em situação de vulnerabilidade. Considera-se também a configuração de um atendimento acolhedor para a população trans jovem por profissionais capacitados para orientação efetiva. Isso pode indicar que a PrEP pode ser uma boa estratégia para a proteção contra o HIV nessa população que já está em alta vulnerabilidade para a infecção.^27,28^ Em 2022, foi lançada a atualização dos Protocolos Clínicos e Diretrizes Terapêuticas para o uso da PrEP, que ampliou a faixa etária para o uso dessa tecnologia para adolescentes com 15 anos ou mais.^29^

Ressalta-se que a experiência de discriminação de gênero referida pelas adolescentes travestis e mulheres trans nos serviços de saúde ou por profissionais desses serviços é uma barreira de acesso e adesão a estratégias de prevenção combinada, como a testagem para o HIV e o uso de PrEP. Mesmo que o presente estudo tenha criado serviços acolhedores e especialmente voltados às jovens trans participantes, as experiências anteriores de discriminação sofridas em outros serviços podem dificultar a quebra da barreira já internalizada pelas adolescentes travestis e mulheres trans para estabelecer vínculo de confiança com os profissionais ou com o próprio serviço.^12,27,30^

Este estudo possuiu algumas limitações. O tamanho amostral, o recrutamento por conveniência, dificulta a aplicação de metodologias mais robustas para a definição da magnitude do efeito entre as variáveis estudadas. Contudo, o tamanho amostral não foi uma barreira, uma vez que a proposta do estudo é ser descritivo, assim como seus resultados se alinham com os demais encontrados na literatura. 

Mesmo que haja motivação para a procura de estratégias de prevenção, a discriminação de gênero, em especial no contexto da atenção em saúde para adolescentes travestis e mulheres trans, é um dos principais empecilhos para o estabelecimento de cuidado de saúde efetivo e ético. Como resultado, o acesso à prevenção ao HIV, a exemplo da testagem regular, o diagnóstico precoce ou o uso de métodos preventivos, a exemplo do acesso ao preservativo ou à PrEP, ficam limitados. Em se tratando de adolescentes, os comportamentos e as atitudes da falta de prevenção poderão perdurar pelo resto da vida.

Faz-se necessário erradicar a discriminação de gênero no âmbito da atenção à saúde e assegurar o acesso adequado para população trans desde a adolescência. São fundamentais: a ampliação de cobertura de saúde para as adolescentes travestis e mulheres trans no Sistema Único de Saúde para além dos ambulatórios especializados; o investimento na criação de demanda através de ações que atraiam as populações mais jovens; e a garantia da vinculação aos serviços de saúde estabelecendo locais acolhedores no Sistema Único de Saúde.
